# Loss of *matK *RNA editing in seed plant chloroplasts

**DOI:** 10.1186/1471-2148-9-201

**Published:** 2009-08-13

**Authors:** Michael Tillich, Vinh Le Sy, Katrin Schulerowitz, Arndt von Haeseler, Uwe G Maier, Christian Schmitz-Linneweber

**Affiliations:** 1 Institut für Biologie, Humboldt Universität zu Berlin, Molekulare Genetik, D-10115 Berlin, Germany; 2 Center for Integrative Bioinformatics Vienna, Max F Perutz Laboratories, University of Vienna, Medical University Vienna, University of Veterinary Medicine Vienna, A-1030 Vienna, Austria; 3 Fachbereich Biologie – Zellbiologie, Philipps-Universität Marburg, Karl-von-Frisch-Str, D-35032 Marburg, Germany; 4 Department of Computer Sciences, College of Technology, Vietnam National University, Hanoi, Vietnam

## Abstract

**Background:**

RNA editing in chloroplasts of angiosperms proceeds by C-to-U conversions at specific sites. Nuclear-encoded factors are required for the recognition of *cis*-elements located immediately upstream of editing sites. The ensemble of editing sites in a chloroplast genome differs widely between species, and editing sites are thought to evolve rapidly. However, large-scale analyses of the evolution of individual editing sites have not yet been undertaken.

**Results:**

Here, we analyzed the evolution of two chloroplast editing sites, *matK*-2 and *matK*-3, for which DNA sequences from thousands of angiosperm species are available. Both sites are found in most major taxa, including deep-branching families such as the nymphaeaceae. However, 36 isolated taxa scattered across the entire tree lack a C at one of the two *matK *editing sites. Tests of several exemplary species from this *in silico *analysis of *matK *processing unexpectedly revealed that one of the two sites remain unedited in almost half of all species examined. A comparison of sequences between editors and non-editors showed that specific nucleotides co-evolve with the C at the *matK *editing sites, suggesting that these nucleotides are critical for editing-site recognition.

**Conclusion:**

(i) Both *matK *editing sites were present in the common ancestor of all angiosperms and have been independently lost multiple times during angiosperm evolution.

(ii) The editing activities corresponding to *matK*-2 and *matK*-3 are unstable.

(iii) A small number of third-codon positions in the vicinity of editing sites are selectively constrained independent of the presence of the editing site, most likely because of interacting RNA-binding proteins.

## Background

Chloroplast RNA metabolism is characterized by extensive RNA processing, including RNA editing. In chloroplasts of angiosperms, RNA editing proceeds by C-to-U base conversions at specific sites, while in chloroplasts of hornworts, many bryophytes and ferns, U-to-C conversions take place as well [1-3]. RNA editing events almost exclusively change codon identities, and usually restore codons conserved during land plant evolution. Mutational analyses of edited codons have demonstrated that editing is essential for protein function *in vivo *[4,5]. The corresponding machinery is nuclear encoded, and recognizes short stretches of sequence immediately upstream of the C to be converted [6].

RNA editing has been found in chloroplasts of all major land plants. To date, there is no evidence for RNA editing in cyanobacteria, the closest prokaryotic relatives of chloroplasts, or in chlorophyte algae, the closest aquatic relatives of land plants. This phylogenetic distribution suggests that chloroplast RNA editing was "invented" close to the root of land plant radiation [3]. Within land plants, the number of chloroplast RNA editing sites per genome differs among species. Bryophytes and ferns may possess several hundred C-to-U as well as U-to-C RNA editing sites [1-3]. The chloroplast genomes of seed plants harbor far fewer (~30) editing sites, and their location varies even between closely related taxa [6]. At least one land plant, the liverwort *Marchantia polymorpha*, apparently contains no RNA editing sites [7], suggesting that, in principle, RNA editing can become lost from a chloroplast genome. An important question is how the species-specific patterns of editing sites – the editotypes – of seed plant chloroplasts evolved. Differences in editotypes between even closely related species, such as *Nicotiana sylvestris*, *Nicotiana tomentosiformis *and other Solanacean relatives, point to a rapid evolution of editing sites [8,9]. A comparison of editing sites between dicot and monocot organelles supports this notion, demonstrating that the speed of editing site evolution equals or exceeds that of third-codon positions [10]. Analyses of selected transcripts from exemplary species over a wide range of land plants have led to similar conclusions [3,11,12].

While these analyses were meant to illuminate the evolution of editing sites, they do not necessarily shed any light on the evolution of the corresponding editing machinery. To date, the only genetically identified essential editing factors are required for editing specific sites and belong to a family of nuclear-encoded RNA binding proteins, the pentatricopeptide repeat proteins (PPR) [13-19]. Most PPR genes are conserved throughout angiosperm evolution [20] and, unlike editing sites, do not rapidly evolve. In fact, in at least five specific cases, specific nuclear activity is retained in a species despite the loss of the corresponding editing site [5,21,22]. If a site-recognition factor is conserved throughout evolution, this should be reflected in the conservation of the corresponding editing-site *cis*-element, an assumption that was supported by a recent analysis of the *psbL *start codon editing site in 28 species, and the *ndhD *start codon editing site in 21 species [12]. In an attempt to understand editing-site evolution at a higher resolution, we took advantage of the thousands of sequences from previous phylogenetic studies that are available for the chloroplast reading frame of the *matK *protein. We analyzed (i) the evolutionary pattern of *matK *editing sites in angiosperm evolution; (ii) the conservation of editing activity in angiosperms; and (iii) the conservation of editing *cis*-elements throughout angiosperm phylogeny.

## Results

### Intrageneric loss of *matK *editing sites in angiosperms

*matK *is a chloroplast gene located within the *trnK *intron that is believed to play a role in RNA splicing of tRNA-K [UUU, [23,24]]. *matK *is an expressed gene [25], and in many monocots, *matK *transcripts are edited at a single site, termed *matK*-1 [26]. We recently identified an additional editing site in Arabidopsis, referred to as *matK*-2, at nucleotide position 706 (codon 236) relative to the start codon [27]. The corresponding editing event leads to a codon change from histidine (CAU) to tyrosine (UAU). Here, we found a third site, *matK*-3, located 70 nucleotides downstream of site 2 that leads to a serine (UCU) to phenylalanine (UUU) codon transition (codon 259, see below).

The rapidly evolving *matK *gene has been a favorite for determining phylogenetic relationships in angiosperms. As a consequence, several thousand *matK *entries covering the entire angiosperm phylogenetic tree have accumulated in Genbank. We obtained and aligned 1255 *matK *sequences from all major angiosperm groups as well as several gymnosperm species, focusing our analysis on determining whether a C or a T was present at these two newly identified editing sites. For phylogenetic analysis, we mapped our findings onto two phylogenetic trees, one for each editing site [[28], see Additional files 1 and 2]. The leaves of the tree represent genera, which can include several species. Because both trees consist predominately of C-containing genera, the most parsimonious assumption is that the common ancestors of all angiosperms had a C at the editing site. In contrast, the gymnosperm taxa analyzed have a T at *matK*-2 and an A at *matK*-3. Whether the site was lost in gymosperms or gained in angiosperms cannot be determined based on our data. We were unable to extend our alignment to more basal embryophyte groups, such as mosses and ferns, due to extreme sequence divergence. Taken together, these data suggest that the *matK*-2 and *matK*-3 editing sites were already present in the ancestor of all angiosperms.

Given that the editing sites are ancestral, we next asked how many times the sites have been lost during angiosperm evolution. We first sought situations in the tree that are indicative of C-to-T transitions within genera. In most cases, all species within a genus share the same editing site. For example, 24 species in the genus *Ceanothus *carry a C at *matK*-2 (see Additional file 3). However, in six of the 298 genera analyzed, there are species that possessed either a C or a T at *matK*-2, suggestive of a recent base transition. Similarly, seven of the genera analyzed include species with either a C or a T at *matK*-3. We call such taxa "mixed genera" (see Additional File 4 – Table S1). Rarely, we also found mixed genera with A- or G-containing species in addition to T- or C-containing species (see Additional File 5 – Table S2). All mixed genera are nested in branches heavily dominated by pure C-containing genera (e.g., see Additional file 3), suggesting that C-losses occurred independently within these genera.

### Frequent and widespread loss of editing sites within larger angiosperm taxa

**Figure 1:**
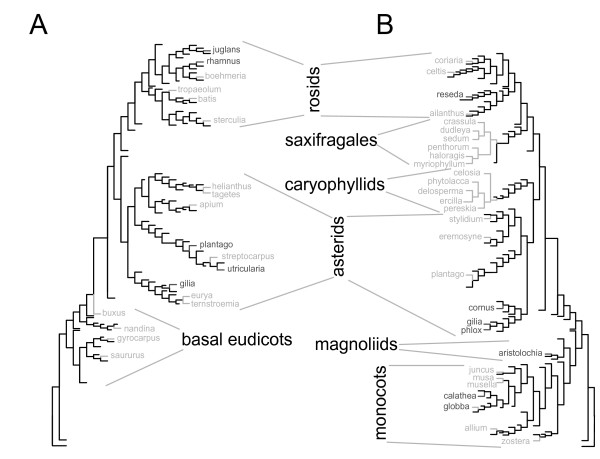
**Multiple losses of *matK *editing sites in angiosperms**. A) Nucleotides found at the *matK*-2 editing site were mapped on a phylogenetic tree encompassing all major angiosperm groups (Soltis et al. 2000). Of the 298 genera investigated, only those that represent independent C-to-T mutations at the editing site are shown (criteria for an independent C-to-T loss are presented in Additional file 3). Additional C-to-T mutations for which independence could not be ascertained are not shown. Branches of the tree without independent C-to-T losses are reduced. The full tree is shown in Additional file 1. Light gray = genera in which all species have a T at the editing site; dark gray = genera containing T-species and C-species. B) Same analysis for *matK*-3; full data is shown in Additional file 2.

If intrageneric loss of editing does occur, it should be also evident on a larger scale. We therefore assessed the distribution of pre-edited (T at the DNA-level) branches of the angiosperm phylogenetic tree that are particularly rich in available *matK *sequences (i.e., Rosids, Saxifragales, Asterids, Caryophyllids, Magnoliids and basal eudicots). Coherent sections of genera without an editing site, for example the Solanaceae/Convolvulaceae, were treated as a unit. We asked whether such pre-edited units are separated from other such units, which would suggest that they had lost editing independently. Only pre-edited units for which sister groups at the next three nodes in the tree contained equal or more than 80% of genera with a C at the editing site were regarded as having independently lost the editing site (see Additional file 3). A- and G-containing genera were not considered. By these criteria, we found evidence for 12 independent losses of edited Cs for *matK*-2 and another 12 for *matK*-3; these were widely distributed throughout the angiosperm tree (see Figure 1 and see Additional file 4 – Table S1). If the intrageneric losses noted above are included here, the number of independent losses for *matK*-2 and *matK*-3 rise to 17 and 19, respectively (Figure 1). Only the asterid genera *Gilia *and *Plantago *have lost both *matK *sites, underscoring that editing-site loss – even that of physically linked sites – is totally independent (Figure 1).

We found no evidence for reversion (i.e. T-to-C back-mutations) for *matK*-2, even within the purely T-containing, large monocot branch. This might indicate the existence of a selective bias towards losing the editing site. It is clear, however, that there are multiple independent losses of the *matK *editing sites throughout angiosperm phylogeny.

### Loss of C-to-U processing in independent branches of the angiosperm tree at *matK*-2 and *matK*-3

The presence of a C at a known editing site is considered good evidence for the presence of a corresponding editing activity. For example, editing events have been successfully predicted by extrapolation from known sites for *Atropa belladonna *and *Pisum sativum *[29,30]. Here, we sequenced amplified cDNA from leaf tissue to investigate RNA editing of *matK*-2 and *matK*-3 in 17 and 14 different angiosperm species, respectively, from disparate sections of the angiosperm phylogenetic tree (see Additional file 6). All species chosen had a C at the *matK*-2 editing sites in the plastid genome. Unexpectedly, we found that *matK*-2 was processed in only seven species (41.2%). In six of these, a C-peak was evident side-by-side with the T-peak in electropherograms. Thus, only a fraction of all transcripts is processed. No editing was detected in RNA samples from the remaining ten species. The loss of editing activity for *matK*-3 was not quite as dramatic; but again, no evidence for editing could be found for two species, and most of the remaining species exhibited only partial editing (see Additional file 6). We call species with a C at the editing site but no detectable editing activity "non-editors", while species that process the C to a U are called "editors". We conclude that editing activities for the *matK *sites have most likely been lost in these species, although the possibility that editing does occur in different tissues under different conditions cannot be ruled out at the moment.

**Figure 2:**
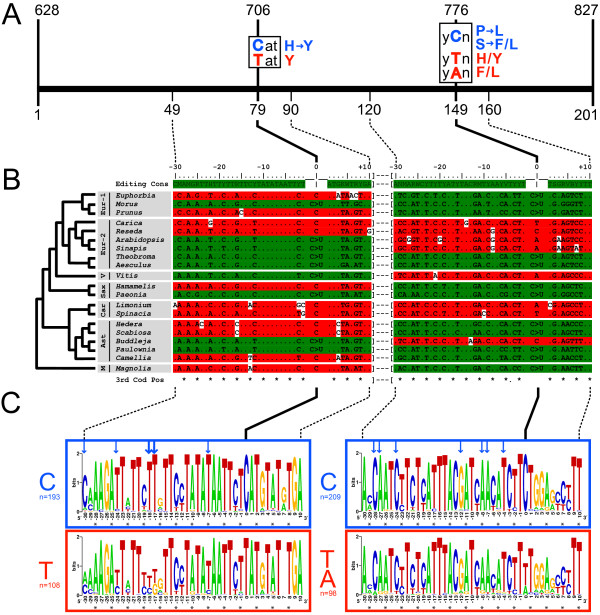
**Analysis of the evolution of *cis*-elements upstream of *matK*-2 and *matK*-3**. A) Schematic representation of the genomic region encompassing the *matK*-2 and *matK*-3 editing sites. Edited Cs and corresponding codon transitions are shown in blue; other bases and corresponding codons at the editing site are shown in red. Numbers above refer to the nucleotide position relative to the first base of the *matK *reading frame in Arabidopsis. This sequence interval was used to generate *matK *alignments. B) Alignment of the sequence interval from -30 to +10 around both *matK *editing sites. Green = species that shows editing at respective *matK *site = "editors" (see Additional file 6); red = species with no detectable editing = "non editors" or with no C at editing site. A consensus sequence was generated based on all edited sequences for each site. Deviations from this consensus are marked in white. Sequences are ordered according to phylogenetic position (Soltis et al, 2000). (Eur = eurosids; V = vitaceae; Sax = saxifragales; Car = cayophyllids; Ast = asterids; M = magnolids.) Third-codon positions are marked with asterisks. C) Analysis of sequence conservation in sequences containing a C at the editing site (C-element; blue border) and in sequences without a C (T-element; red border). Sequences from n different genera were aligned and analyzed using the WebLogo software. Note that n includes one species from each genus in the *matK *trees shown in Additional files 1 and 2, and not just those analyzed in B. Residues exhibiting differential conservation are marked with blue arrows. The two most variable residues are marked with bold arrows. Third-codon positions are marked with asterisks.

To understand the phylogenetic distribution of the underlying RNA editing activities, we mapped our results on a phylogenetic tree (Figure 2B). Editing activities are found at widely separated positions of this tree. For example, editors and non-editors for *matK*-2 are found both in the eurosids I and the eurosids II. Similarly, *matK*-2 editors and non-editors are also present side-by-side in lamiids and campanulids within the asterid clade. This situation is repeated for *matK*-3, where the two species that have lost editing activity are from separate larger taxa: *Reseda *from the rosids and *Buddleja *from the asterids. Taken together with the ancestral nature of the *matK *editing sites, noted above, these findings argue for multiple independent losses of the editing activities.

To investigate whether these losses are reflected in the corresponding *cis*-elements, we generated a consensus sequence for all plants capable of editing and compared it with sequences from the non-editing plants (Figure 2A). We found that almost all non-editors contain one or multiple deviations from the consensus sequence deduced from the set of editors, suggesting a correlation between the loss of the editing activity and the evolutionary degeneration of the *cis*-element.

### Conservation of putative recognition elements for a *matK*-2 *trans*-acting factor

Editing sites are recognized by RNA binding proteins that bind sequence elements immediately upstream of the C-residue to be edited. As long as binding and editing processes continue to occur, selection is expected to act to preserve these *cis*-elements. By contrast, it is expected that the loss of editing would be accompanied by the loss of conservation of *trans*-acting factor binding-site sequences. To identify such sequence elements, we prepared separate alignments of sequences containing a C and those containing a T at the *matK *editing sites (henceforth called C-elements and T-elements, respectively). To avoid a bias toward species-rich genera, we randomly selected one sequence from each genus. The sequences were aligned and analysed using the WebLogo software [31] in order to visualize sequence conservation, and alignments were scored from position -30 to +10, where the editing site is +1. Figure 2C shows a comparison of the conservation of this sequence window between C- and T-containing *matK*-2 and *matK*-3 sites. The following three conservational classes for individual nucleotides can be distinguished:

(i) Nucleotide positions that are conserved in both C- and T-elements; for example, at positions -27 to -25, -6 to -4 and +8 to +10 relative to *matK*-2, and -17 to -15 upstream of *matK*-3. These include third-codon positions (e.g., *matK*-2 positions -25 and -4; *matK*-3 positions -14 and -20), for which other evolutionary constraints apart from coding must be responsible.

(ii) Nucleotide positions that are variable in both T- and C-elements, mostly third-codon positions (e.g., *matK*-2 positions -22, -19 and -16).

(iii) Nucleotide positions that are conserved only in editors. For *matK*-2, we found five highly conserved positions at -7, -17, -18, -24 and -30 of C-elements, whereas the corresponding positions in T-elements are much more variable. Conservation of the dominant base at these positions is 100% (-7, -17), 96% (-18), 93% (-24) and 88% (-30) in C-elements, but only 83% (-7), 55% (-17), 62% (-18, -24) and 45% (-30) in T-elements (see also arrows in Figure 2C). Notably, the highly conserved T at base -7 in C-elements is at a third-codon position. An analysis of a longer stretch of sequence upstream of the *matK*-2 editing site revealed that differential conservation terminates at position -30, and thus coincides with the location of the expected *cis*-element for editing (data not shown). For *matK*-3, such differential conservation between C-elements and T-elements is less pronounced, although differences exist at positions -4, -7, -8, -12, -24, -27 and -28.

These comparisons demonstrate that selected upstream bases and the C at the editing site have co-evolved. Furthermore, high conservation of several third-codon positions in **both **C- and T-elements suggests a selective force that is independent of both amino-acid coding and the editing site at these positions. Finally, a stronger conservation of bases in T-sites relative to C-sites was not observed for *matK*-2 or *matK*-3, supporting the conclusion that the observed conservation bias is functionally linked to the editing site.

## Discussion

### Loss of *matK *editing sites in angiosperm evolution

It is impossible to clearly infer the loss or gain of an editing site by examining a limited set of sequences because any conclusion drawn ultimately relies on only one informative site: the editing site itself. Thus, an understanding of the evolutionary history of RNA editing sites requires an analysis of a large set of related sequences. We have therefore investigated the evolutionary behavior of two editing sites and their presumptive *cis*-elements in the *matK *gene, an approach that allows us to track the editing site throughout a continuum of related angiosperm sequences. Our results show that C dominates the phylogenetic trees for both *matK-*2 and *matK-*3 sites; thus, the most parsimonious explanation is that both editing sites were already present in the ancestor of all angiosperms. A closer analysis of the distribution of species and genera lacking a C at the editing sites suggests that the C at both *matK *sites was lost independently on multiple occasions. These data support earlier work suggesting that ancient angiosperms contained high numbers of editing sites that were lost independently in separate taxonomic branches during angiosperm evolution [32]. Our results are also consistent with a study on the evolution of mitochondrial editing that described multiple independent losses of editing sites in selected monocot taxa [33]. Importantly, these studies collectively explain the variability of editotypes among angiosperms species solely by invoking loss of editing sites, and do not require a presumption of balanced loss and gain of sites. Although preliminary, our results show no evidence for re-acquisition of *matK *editing sites, as exemplified for *matK-2 *in the purely T-carrying monocots. This suggests that at least these two sites, and by extrapolation, possibly all plastid-editing sites, are "on the way out".

### Loss of *matK *editing activity in angiosperm evolution

An unexpected finding of this study is the loss or reduction in RNA editing in many species despite the presence of a C at the editing site. Reduced editing can either be caused by the degeneration of nuclear-encoded editing factors or plastidial *cis*-elements that direct the editing machinery. Based on the assumption that the *matK *editing activities are ancient (like the sites themselves; see above), we argue for multiple independent losses of editing at both sites during angiosperm evolution. Editing of *matK*-3 leads to a change in the codons that results in incorporation of very different amino acids: serine and phenylalanine. Given the nature of this difference, it is remarkable that *Buddleja *and *Reseda *tolerate the loss of this editing event. By contrast, the rather minor physico-chemical change provided by an H-to-Y amino acid transition mediated by *matK*-2 editing might be less critical for protein function. Among the codon transitions caused by chloroplast RNA editing, this codon transition is one of the rarest and therefore might not be as important for protein function as the much more frequent S-to-L or P-to-L transitions. The MatK protein may tolerate both amino acids, in which case the loss of RNA editing would have only limited consequences for protein function. If it is indeed selectively neutral, the frequent loss of C observed here might be specific for *matK *editing and thus not generalizable to truly essential RNA-editing sites. However, the fact that several independent C-to-T mutations, but no T-to-C back-mutations, are observed at both sites suggests that the edited amino acid is under positive selection. A reduction or a loss of editing could generate such a selective pressure for a C-to-T mutation and lead to the elimination of an editing site. Therefore, our results suggest that a decay in editing efficiency precedes the loss of editing sites, as proposed by Schields and Wolfe [10]. Whether a degeneration of editing factors or their corresponding *cis*-elements is responsible for the reductions in editing efficiency observed here cannot be determined by our analyses because the reductions co-occur with mutations in *cis*-elements. Notably, chloroplast genomes display an enhanced genetic drift and accumulate mildly deleterious point mutations [34]. *MatK *is one of the most rapidly diverging plastidial genes and exhibits a relatively high rate of degeneration [25]. Therefore, we speculate that it is rather the degeneration of *cis*-elements that leads to the observed reduction in editing efficiencies, which in turn generates the selective pressure responsible for the frequent losses of editing sites by C-to-T mutations.

### *Cis*-elements of *matK *editing sites are under multiple selective constraints

We have carried out a phylogenetic analysis of predicted *matK*-2 and *matK*-3 *cis*-elements in order to identify a putative conserved binding site for the corresponding (unknown) *trans*-acting factor(s). Many bases in these *cis*-elements are conserved in both C- and T-elements, mostly due to coding constraints, but several third-codon positions are also conserved. This could mean that selection is acting on all analyzed sequences, no matter which base is present at the editing site. If this selection is sufficiently stringent and acts on all bases, there should be no conservation bias towards C-elements. Irrespective of RNA editing function, a factor binding to this sequence, either on DNA or RNA, could provide such a selective force.

Our analysis uncovered five bases that are highly conserved in sequences containing the *matK*-2 editing site, but not in those lacking the site. A co-evolution of these bases with the editing site most likely reflects a function for these bases in editing-site processing or recognition. Such co-evolving nucleotides have recently been identified for two chloroplast editing sites, albeit in a much smaller taxon sampling [12]. Intriguingly, *in vitro *studies have demonstrated that bases within such *cis*-elements have strikingly unequal impacts on RNA editing [35,36]. For example, mutations of the -2 and -3 nucleotides of the *psbE *editing site led to a pronounced reduction in editing efficiency *in vitro*, while mutations at the adjacent -4/-5 and +2/+3 sites had only minor effects [35]. Similar major effects of single bases on editing have also been observed *in vivo *[37]. The position-specific inhibition of activity is not reflected in a similar inhibition of binding: all mutated versions of *cis*-elements appear to be equally good binding sites for (unknown) *trans*-acting factors [35]. Thus, the bases co-conserved with the *matK*-2 editing site might be important for RNA editing activity, while their role in binding of *trans*-acting factors could be minor. In other words, the same RNA-binding protein that attaches to C-elements might also bind to T-elements. Such a factor could perform an additional function (or functions) unrelated to editing, and conserved bases could be important for such secondary function(s) of the editing factor. This would explain why bases are conserved at several third-codon positions in both C- and T-elements. Recently, PPR proteins have been identified as editing factors [13,14]. Although these proteins are highly conserved between rice and Arabidopsis [20], their target Cs are not: only nine editing positions are conserved between rice [38] and Arabidopsis [39]. For instance, the *ndhD *editing site, served by CRR21 in Arabidopsis, is lacking in rice; however, despite absence of the corresponding site, an orthologous protein can be readily identified (data not shown). The simplest explanation is that these factors may be involved in editing, but also serve additional, evolutionarily more stable functions. Our finding that many species carrying a C at the editing site lack editing activity might indicate that the corresponding factors have been lost. Such a loss-of-factor scenario would be consistent with several studies that demonstrated that transfer of editing sites from one species to another often leads to a failure to process the heterologous site, i.e. are indicative of a loss of the corresponding editing factor [4,21,40]. Three observations, however, speak against this simple loss-of-factor scenario: (i) several transferred sites are heterologously edited [21,22]; (ii) PPRs, the *bona fide *editing factors, are conserved in angiosperms and are thus not reflective of editotype variability; (iii) our phylogenetic analysis uncovered sequence conservation in *cis*-elements at third-codon positions, not only in editors but also in non-editors and T-carriers. These considerations lead us to hypothesize that the factors are conserved and still bind *cis*-elements, but their editing activity is compromised because of mutations that disrupt protein structure/function or subtly alter RNA binding properties Determining whether known editing factors have additional functions and whether these functions are conserved in species that are devoid of the cognate editing site would be of great value in testing this hypothesis.

## Conclusion

In this paper, we focused on the evolution of chloroplast editing sites in angiosperms. We demonstrate for the entire angiosperm radiation that editing sites have been lost multiple independent times. Our data also uncover a surprisingly frequent reduction or loss of the corresponding activity in selected taxa. Finally, this large-scale analysis helped to detect nucleotides with close co-evolutionary ties to the edited C. The additional finding that evolutionary conservation of third-codon positions can be detected even in the absence of an edited C supports the idea that interactions of *trans*-acting factors with sequence elements surrounding editing sites take also place for reasons other than RNA editing.

## Methods

### Plant material

All leaf material was collected in the Botanical Garden of Marburg, Germany.

### RNA preparation/RT-PCR analysis

RNA extraction was performed using the TRIzol Reagent according to the supplier's instructions, or by a cetyltrimethylammonium bromide (CTAB)-based method as described by Zeng and Yang [41]. Five to eight micrograms RNA were treated with DNaseI (Roche, 40 u, 1 h, 37°C) to remove any DNA contamination. The RNA was then purified by two phenol/chloroform extractions, one chloroform extraction and an ethanol/salt precipitation step. cDNA sequences were amplified by PCR (Qiagen) after reverse transcription using the Omniscript RT-Kit (Qiagen) employing random hexamers, or the One-Step-RT-PCR Kit (Qiagen). An aliquot of RNA that was not reverse transcribed served as a control PCR template for DNA contamination. Total cellular DNA was extracted using a standard CTAB-based method.

### Oligonucleotides

The following oligonucleotides (5'>3') were used to amplify *matK*-2 and *matK*-3 sequences from DNA or cDNA: rctccttctttgcatttattgcg (matk.for.a), gctccttctttgcatttattgag (matk.for.b), gcctcttctttgcatttattgcg (matk.for.c), gcctcttctttgcatttattacg (matk.for.d), ccttcttctttacattttttacg, (matk.for.e), acctcttctttgcatttattaag (matk.for.f), catgaaaggatccttgaacaacc (matk.rev.z), catgaagagatcctcgaggaacc (matk.rev.y), agagaarggktctttgaaaagcc (matk.rev.x), awgaaaagkatctttgaaaaacc (matk.rev.w), catgaaaggatccttsaacaaca (matk.rev.v), tatgaaaggattcttgaacaaac (matk.rev.u) and cgcaaaaggatccttaagtaacc (matk.rev.t).

### Sequence analysis

PCR products were purified using the NucleoSpin Extract II-Kit (Macherey and Nagel) and sequenced using DYEnamic ET chemistry (GE Healthcare) according to the supplier's instructions. The products of the sequencing reactions were analyzed on an ABI 377 automated sequencer (Applied Biosystems) according to the manufacturer's instructions.

### Phylogenetic analysis

A total of 1255 *matK *sequences covering 298 major angiosperm genera were obtained from GenBank. All genera represent leaves of phylogenetic trees constructed by Soltis et al. [[28], see Additional files 1 and 2]. Sequences were aligned by ClustalW using default parameters [42] resulting in an alignment of 3455 nt. A 201-bp sequence window comprising the editing sites *matK*-2 and *matK*-3 was extracted from this alignment for analysis.

## Authors' contributions

MT conceived of the study, carried out the cDNA analyses together with KS and participated in the sequence alignment. VLS and AvH generated the sequence alignment and mapped the results for *matK *editing sites on the phylogenetic tree. CSL participated in the design of the study and wrote the draft manuscript. All authors contributed to writing the manuscript and drawing the figures, and all approved the final version.

## Supplementary Material

Additional file 1 **Evolution of *matK*-2 editing sites in angiosperms**. This phylogenetic tree shows all detected losses for *matK *editing site 2 during angiosperm evolution. Click here for file

Additional file 2 **Evolution of *matK*-3 editing sites in angiosperms**. This phylogenetic tree shows all detected losses for *matK *editing site 3 during angiosperm evolution. Click here for file

Additional file 3 **Examples of *matK*-2 editing sites lost during Angiosperm evolution**. An excerpt of the phylogenetic tree shown in Additional file 1 labeled to demonstrate the method used to evaluate losses of editing sites during *matK *evolution in angiosperms. Click here for file

Additional file 4 **List of independent C-to-T losses in angiosperm evolution at *matK *editing sites**. A table listing all C-to-T losses at *matK *editing sites identified in this study based on the analysis of the phylogenetic trees shown in Additional files 1 and 2. Click here for file

Additional file 5 **List of independent C-to-A and C-to-G losses in angiosperm evolution at the *matK*-3 editing site**. A table listing all C-to-A and C-to-G mutations at *matK *editing sites identified in this study based on the analysis of the phylogenetic trees shown in Additional files 1 and 2. Click here for file

Additional file 6 **Analysis of *matK*-2 and *matK*-3 editing in selected species**. Excerpts from cDNA sequencing electropherograms are shown to demonstrate the extent of editing in selected angiosperm species. Click here for file
