# Supplementary material for: Loss of matK RNA editing in seed plant chloroplasts
Source: BMC Evol Biol. 2009 Aug 13;9:201. doi: 10.1186/1471-2148-9-201 (PMC2744683; doi:10.1186/1471-2148-9-201)
Supplement: Additional file 1 — Evolution of matK-2 editing sites in angiosperms. This phylogenetic tree shows all detected losses for matK editing site 2 during angiosperm evolution. [file 1471-2148-9-201-S1.pdf]

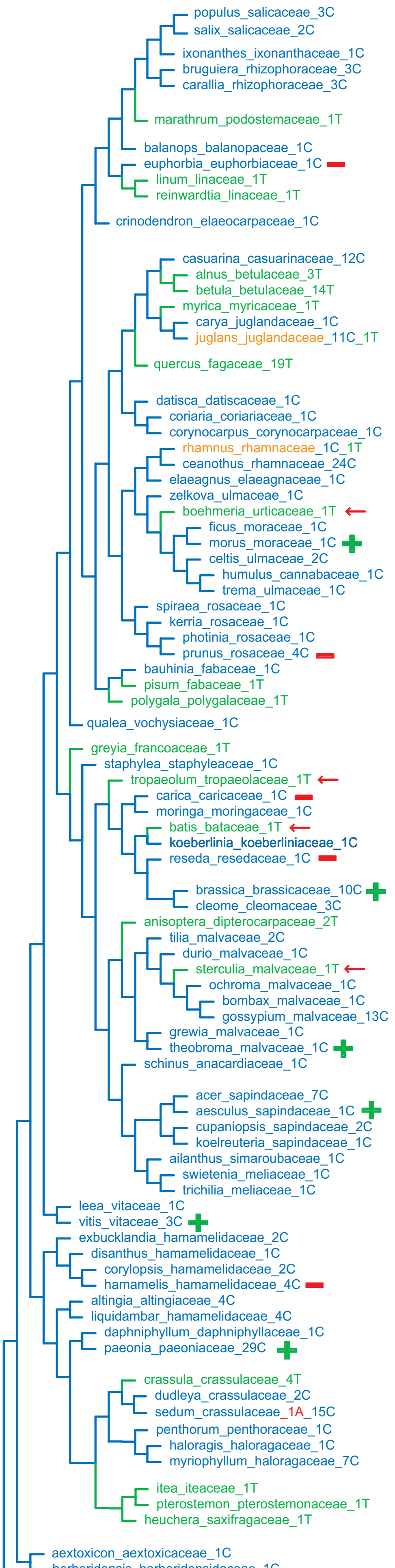
populus_salicaceae_3C
salix_salicaceae_2C
ixonanthes_ixonanthaceae_1C
bruguiera_rhizophoraceae_3C
carallia_rhizophoraceae_3C
marathrum_podostemaceae_1T
balanops_balanopaceae_1C
euphorbia_euphorbiaceae_1C
linum_linaceae_1T
reinwardtia_linaceae_1T
crinodendron_elaeocarpaceae_1C
casuarina_casuarinaceae_12C
alnus_betulaceae_3T
betula_betulaceae_14T
myrica_myricaceae_1T
carya_juglandaceae_1C
juglans_juglandaceae_11C_1T
quercus_fagaceae_19T
datisca_datiscaceae_1C
coriaria_coriariaceae_1C
corynocarpus_corynocarpaceae_1C
rhamnus_rhamnaceae_1C_1T
ceanothus_rhamnaceae_24C
elaeagnus_elaeagnaceae_1C
zelkova_ulmaceae_1C
boehmeria_urticaceae_1T
ficus_moraceae_1C
morus_moraceae_1C
celtis_ulmaceae_2C
humulus_cannabaceae_1C
trema_ulmaceae_1C
spiraea_rosaceae_1C
kerria_rosaceae_1C
photinia_rosaceae_1C
prunus_rosaceae_4C
bauhinia_fabaceae_1C
pisum_fabaceae_1T
polygala_polygalaceae_1T
qualea_vochysiaceae_1C
greyia_francoaceae_1T
staphylea_staphyleaceae_1C
tropaeolum_tropaeolaceae_1T
carica_caricaceae_1C
moringa_moringaceae_1C
batis_bataceae_1T
koeberlinia_koeberliniaceae_1C
reseda_resedaceae_1C
brassica_brassicaceae_10C
cleome_cleomaceae_3C
anisoptera_dipterocarpaceae_2T
tilia_malvaceae_2C
durio_malvaceae_1C
sterculia_malvaceae_1T
ochroma_malvaceae_1C
bombax_malvaceae_1C
gossypium_malvaceae_13C
grewia_malvaceae_1C
theobroma_malvaceae_1C
schinus_anacardiaceae_1C
acer_sapindaceae_7C
aesculus_sapindaceae_1C
cupaniopsis_sapindaceae_2C
koelreuteria_sapindaceae_1C
ailanthus_simaroubaceae_1C
swietenia_meliaceae_1C
trichilia_meliaceae_1C
leea_vitaceae_1C
vitis_vitaceae_3C
exbucklandia_hamamelidaceae_2C
disanthus_hamamelidaceae_1C
corylopsis_hamamelidaceae_2C
hamamelis_hamamelidaceae_4C
altingia_altingiaceae_4C
liquidambar_hamamelidaceae_4C
daphniphyllum_daphniphyllaceae_1C
paeonia_paeoniaceae_29C
crassula_crassulaceae_4T
dudleya_crassulaceae_2C
sedum_crassulaceae_1A_15C
penthorum_penthoraceae_1C
haloragis_haloragaceae_1C
myriophyllum_haloragaceae_7C
itea_iteaceae_1T
pterostemon_pterostemonaceae_1T
heuchera_saxifragaceae_1T
aextoxicon_aextoxicaceae_1C

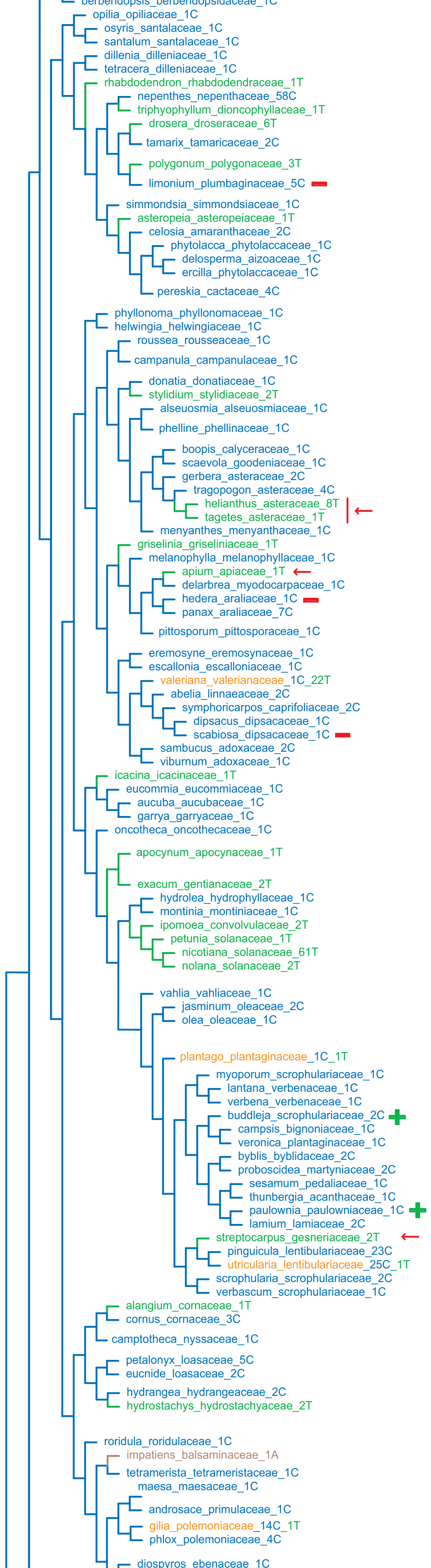
berberidopsis_berberidopsidaceae_1C
opilia_opiliaceae_1C
osyris_santalaceae_1C
santalum_santalaceae_1C
dillenia_dilleniaceae_1C
tetracera_dilleniaceae_1C
rhabdodendron_rhabdodendraceae_1T
nepenthes_nepenthaceae_58C
triphyophyllum_dioncophyllaceae_1T
drosera_droseraceae_6T
tamarix_tamaricaceae_2C
polygonum_polygonaceae_3T
limonium_plumbaginaceae_5C
simmondsia_simmondsiaceae_1C
asteropeia_asteropeiaceae_1T
celosia_amaranthaceae_2C
phytolacca_phytolaccaceae_1C
delosperma_aizoaceae_1C
ercilla_phytolaccaceae_1C
pereskia_cactaceae_4C
phyllonoma_phyllonomaceae_1C
helwingia_helwingiaceae_1C
roussea_rousseaceae_1C
campanula_campanulaceae_1C
donatia_donatiaceae_1C
stylidium_stylidiaceae_2T
alseuosmia_alseuosmiaceae_1C
phelline_phellinaceae_1C
boopis_calyceraceae_1C
scaevola_goodeniaceae_1C
gerbera_asteraceae_2C
tragopogon_asteraceae_4C
helianthus_asteraceae_8T
tagetes_asteraceae_1T
menyanthes_menyanthaceae_1C
griselinia_griseliniaceae_1T
melanophylla_melanophyllaceae_1C
apium_apiaceae_1T
delarbrea_myodocarpaceae_1C
hedera_araliaceae_1C
panax_araliaceae_7C
pittosporum_pittosporaceae_1C
eremosyne_eremosynaceae_1C
escallonia_escalloniaceae_1C
valeriana_valerianaceae_1C_22T
abelia_linnaeaceae_2C
symphoricarpos_caprifoliaceae_2C
dipsacus_dipsacaceae_1C
scabiosa_dipsacaceae_1C
sambucus_adoxaceae_2C
viburnum_adoxaceae_1C
icacina_icacinaceae_1T
eucommia_eucommiaceae_1C
aucuba_aucubaceae_1C
garrya_garryaceae_1C
oncotheca_oncothecaceae_1C
apocynum_apocynaceae_1T
exacum_gentianaceae_2T
hydrolea_hydrophyllaceae_1C
montinia_montiniaceae_1C
ipomoea_convolvulaceae_2T
petunia_solanaceae_1T
nicotiana_solanaceae_61T
nolana_solanaceae_2T
vahlia_vahliaceae_1C
jasminum_oleaceae_2C
olea_oleaceae_1C
plantago_plantaginaceae_1C_1T
myoporum_scrophulariaceae_1C
lantana_verbenaceae_1C
verbena_verbenaceae_1C
buddleja_scrophulariaceae_2C
campsis_bignoniaceae_1C
veronica_plantaginaceae_1C
byblis_byblidaceae_2C
proboscidea_martyniaceae_2C
sesamum_pedaliaceae_1C
thunbergia_acanthaceae_1C
paulownia_paulowniaceae_1C
lamium_lamiaceae_2C
streptocarpus_gesneriaceae_2T
pinguicula_lentibulariaceae_23C
utricularia_lentibulariaceae_25C_1T
scrophularia_scrophulariaceae_2C
verbascum_scrophulariaceae_1C
alangium_cornaceae_1T
cornus_cornaceae_3C
camptotheca_nyssaceae_1C
petalonyx_loasaceae_5C
eucnide_loasaceae_2C
hydrangea_hydrangeaceae_2C
hydrostachys_hydrostachyaceae_2T
roridula_roridulaceae_1C
impatiens_balsaminaceae_1A
tetramerista_tetrameristaceae_1C
maesa_maesaceae_1C
androsace_primulaceae_1C
gilia_polemoniaceae_14C_1T
phlox_polemoniaceae_4C
diospyros_ebenaceae_1C

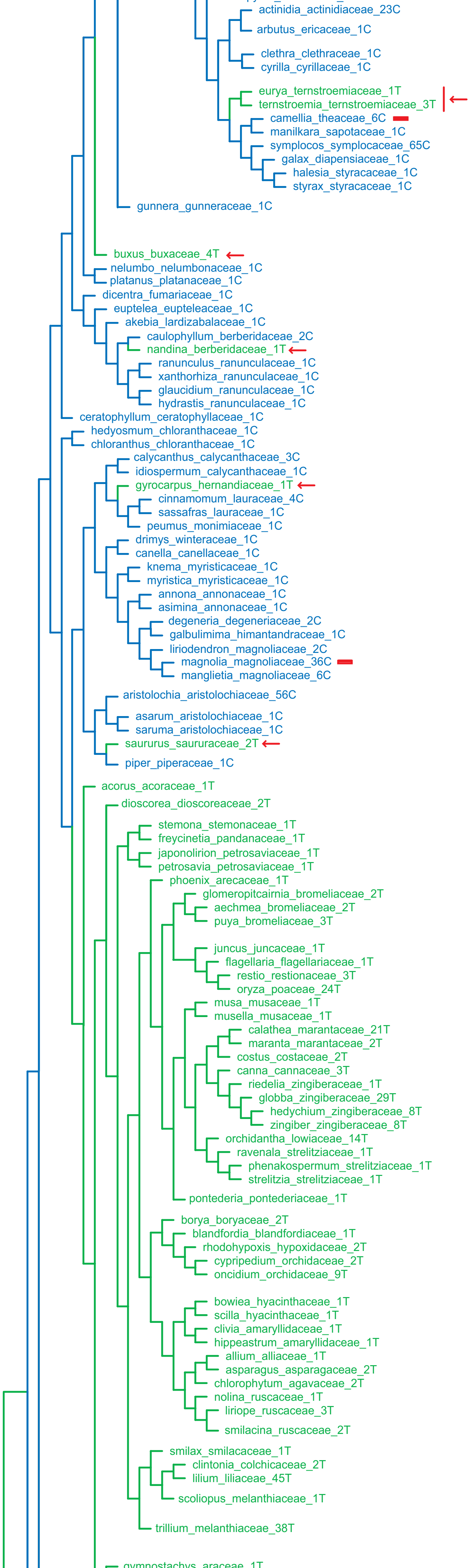
actinidia_actinidiaceae_23C
arbutus_ericaceae_1C
clethra_clethraceae_1C
cyrilla_cyrillaceae_1C
eurya_ternstroemiaceae_1T
ternstroemia_ternstroemiaceae_3T
camellia_theaceae_6C
manilkara_sapotaceae_1C
symplocos_symplocaceae_65C
galax_diapensiaceae_1C
halesia_styracaceae_1C
styrax_styracaceae_1C
gunnera_gunneraceae_1C
buxus_buxaceae_4T
nelumbo_nelumbonaceae_1C
platanus_platanaceae_1C
dicentra_fumariaceae_1C
euptelea_eupteleaceae_1C
akebia_lardizabalaceae_1C
caulophyllum_berberidaceae_2C
nandina_berberidaceae_1T
ranunculus_ranunculaceae_1C
xanthorhiza_ranunculaceae_1C
glaucidium_ranunculaceae_1C
hydrastis_ranunculaceae_1C
ceratophyllum_ceratophyllaceae_1C
hedyosmum_chloranthaceae_1C
chloranthus_chloranthaceae_1C
calycanthus_calycanthaceae_3C
idiospermum_calycanthaceae_1C
gyrocarpus_hernandiaceae_1T
cinnamomum_lauraceae_4C
sassafras_lauraceae_1C
peumus_monimiaceae_1C
drimys_winteraceae_1C
canella_canellaceae_1C
knema_myristicaceae_1C
myristica_myristicaceae_1C
annona_annonaceae_1C
asimina_annonaceae_1C
degeneria_degeneriaceae_2C
galbulimima_himantandraceae_1C
liriodendron_magnoliaceae_2C
magnolia_magnoliaceae_36C
manglietia_magnoliaceae_6C
aristolochia_aristolochiaceae_56C
asarum_aristolochiaceae_1C
saruma_aristolochiaceae_1C
saururus_saururaceae_2T
piper_piperaceae_1C
acorus_acoraceae_1T
dioscorea_dioscoreaceae_2T
stemona_stemonaceae_1T
freycinetia_pandanaceae_1T
japonolirion_petrosaviaceae_1T
petrosavia_petrosaviaceae_1T
phoenix_arecaceae_1T
glomeropitcairnia_bromeliaceae_2T
aechmea_bromeliaceae_2T
puya_bromeliaceae_3T
juncus_juncaceae_1T
flagellaria_flagellariaceae_1T
restio_restionaceae_3T
oryza_poaceae_24T
musa_musaceae_1T
musella_musaceae_1T
calathea_marantaceae_21T
maranta_marantaceae_2T
costus_costaceae_2T
canna_cannaceae_3T
riedelia_zingiberaceae_1T
globba_zingiberaceae_29T
hedychium_zingiberaceae_8T
zingiber_zingiberaceae_8T
orchidantha_lowiaceae_14T
ravenala_strelitziaceae_1T
phenakospermum_strelitziaceae_1T
strelitzia_strelitziaceae_1T
pontederia_pontederiaceae_1T
borya_boryaceae_2T
blandfordia_blandfordiaceae_1T
rhodohypoxis_hypoxidaceae_2T
cypripedium_orchidaceae_2T
oncidium_orchidaceae_9T
bowiea_hyacinthaceae_1T
scilla_hyacinthaceae_1T
clivia_amaryllidaceae_1T
hippeastrum_amaryllidaceae_1T
allium_alliaceae_1T
asparagus_asparagaceae_2T
chlorophytum_agavaceae_2T
nolina_ruscaceae_1T
liriope_ruscaceae_3T
smilacina_ruscaceae_2T
smilax_smilacaceae_1T
clintonia_colchicaceae_2T
lilium_liliaceae_45T
scoliopus_melanthiaceae_1T
trillium_melanthiaceae_38T

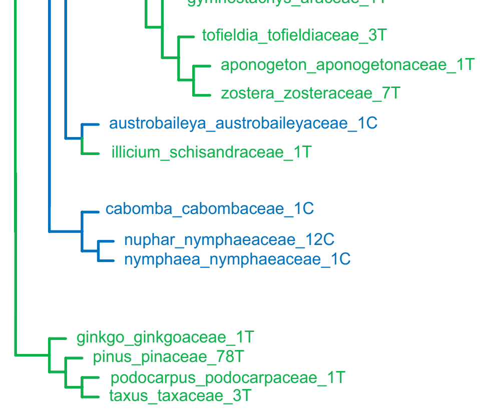

Evolution of *matK*-2 editing sites in angiosperms

Nucleotides found at the *matK*-2 editing site in angiosperms were mapped on a phylogenetic tree (Soltis et al. 2000). Four gymnosperms were used as an outgroup. Genus and family names are follo - wed by a number indicating the number of species carrying a C or a T at the editing site (blue = purely C-containing lineages; T = purely T-containing lineages; brown = purely A-containing lineages; orange = mixed lineages). Several species were tested experimentally for processing of *matK*-2. (+/- = selected species of this genus does/does not edit site; red arrows = lineages that have independently lost the C at *matK*-2.)
