# Supplementary material for: Loss of matK RNA editing in seed plant chloroplasts
Source: BMC Evol Biol. 2009 Aug 13;9:201. doi: 10.1186/1471-2148-9-201 (PMC2744683; doi:10.1186/1471-2148-9-201)
Supplement: Additional file 2 — Evolution of matK-3 editing sites in angiosperms. This phylogenetic tree shows all detected losses for matK editing site 3 during angiosperm evolution. [file 1471-2148-9-201-S2.pdf]

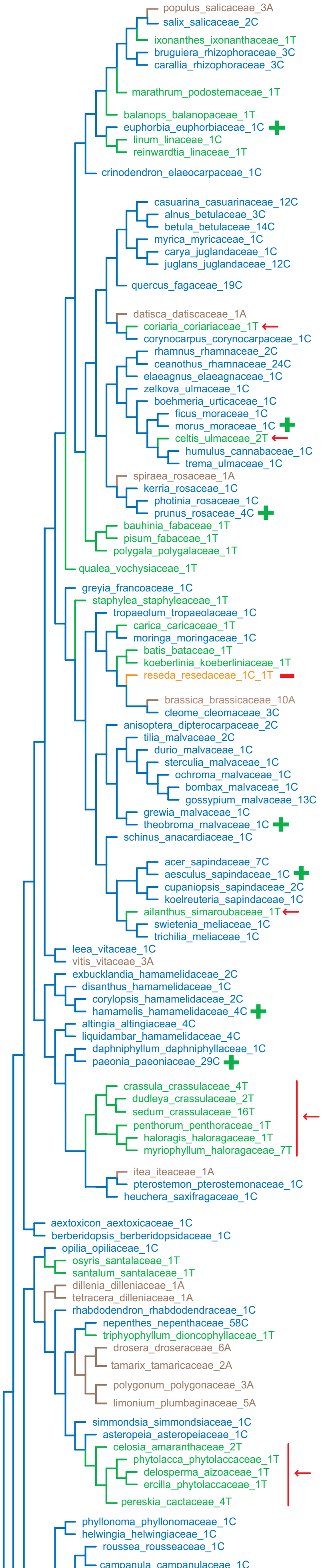
populus_salicaceae_3A
salix_salicaceae_2C
ixonanthes_ixonanthaceae_1T
bruguiera_rhizophoraceae_3C
carallia_rhizophoraceae_3C
marathrum_podostemaceae_1T
balanops_balanopaceae_1T
euphorbia_euphorbiaceae_1C
linum_linaceae_1C
reinwardtia_linaceae_1T
crinodendron_elaeocarpaceae_1C
casuarina_casuarinaceae_12C
alnus_betulaceae_3C
betula_betulaceae_14C
myrica_myricaceae_1C
carya_juglandaceae_1C
juglans_juglandaceae_12C
quercus_fagaceae_19C
datisca_datiscaceae_1A
coriaria_coriariaceae_1T
corynocarpus_corynocarpaceae_1C
rhamnus_rhamnaceae_2C
ceanothus_rhamnaceae_24C
elaeagnus_elaeagnaceae_1C
zelkova_ulmaceae_1C
boehmeria_urticaceae_1C
ficus_moraceae_1C
morus_moraceae_1C
celtis_ulmaceae_2T
humulus_cannabaceae_1C
trema_ulmaceae_1C
spiraea_rosaceae_1A
kerria_rosaceae_1C
photinia_rosaceae_1C
prunus_rosaceae_4C
bauhinia_fabaceae_1T
pisum_fabaceae_1T
polygala_polygalaceae_1T
qualea_vochysiaceae_1T
greyia_francoaceae_1C
staphylea_staphyleaceae_1T
tropaeolum_tropaeolaceae_1C
carica_caricaceae_1T
moringa_moringaceae_1C
batis_bataceae_1T
koeberlinia_koeberliniaceae_1T
reseda_resedaceae_1C_1T
brassica_brassicaceae_10A
cleome_cleomaceae_3C
anisoptera_dipterocarpaceae_2C
tilia_malvaceae_2C
durio_malvaceae_1C
sterculia_malvaceae_1C
ochroma_malvaceae_1C
bombax_malvaceae_1C
gossypium_malvaceae_13C
grewia_malvaceae_1C
theobroma_malvaceae_1C
schinus_anacardiaceae_1C
acer_sapindaceae_7C
aesculus_sapindaceae_1C
cupaniopsis_sapindaceae_2C
koelreuteria_sapindaceae_1C
ailanthus_simaroubaceae_1T
swietenia_meliaceae_1C
trichilia_meliaceae_1C
leea_vitaceae_1C
vitis_vitaceae_3A
exbucklandia_hamamelidaceae_2C
disanthus_hamamelidaceae_1C
corylopsis_hamamelidaceae_2C
hamamelis_hamamelidaceae_4C
altingia_altingiaceae_4C
liquidambar_hamamelidaceae_4C
daphniphyllum_daphniphyllaceae_1C
paeonia_paeoniaceae_29C
crassula_crassulaceae_4T
dudleya_crassulaceae_2T
sedum_crassulaceae_16T
penthorum_penthoraceae_1T
haloragis_haloragaceae_1T
myriophyllum_haloragaceae_7T
itea_iteaceae_1A
pterostemon_pterostemonaceae_1C
heuchera_saxifragaceae_1C
aextoxicon_aextoxicaceae_1C
berberidopsis_berberidopsidaceae_1C
opilia_opiliaceae_1C
osyris_santalaceae_1T
santalum_santalaceae_1T
dillenia_dilleniaceae_1A
tetracera_dilleniaceae_1A
rhabdodendron_rhabdodendraceae_1C
nepenthes_nepenthaceae_58C
triphyophyllum_dioncophyllaceae_1T
drosera_droseraceae_6A
tamarix_tamaricaceae_2A
polygonum_polygonaceae_3A
limonium_plumbaginaceae_5A
simmondsia_simmondsiaceae_1C
asteropeia_asteropeiaceae_1C
celosia_amaranthaceae_2T
phytolacca_phytolaccaceae_1T
delosperma_aizoaceae_1T
ercilla_phytolaccaceae_1T
pereskia_cactaceae_4T
phyllonoma_phyllonomaceae_1C
helwingia_helwingiaceae_1C
roussea_rousseaceae_1C
campanula_campanulaceae_1C

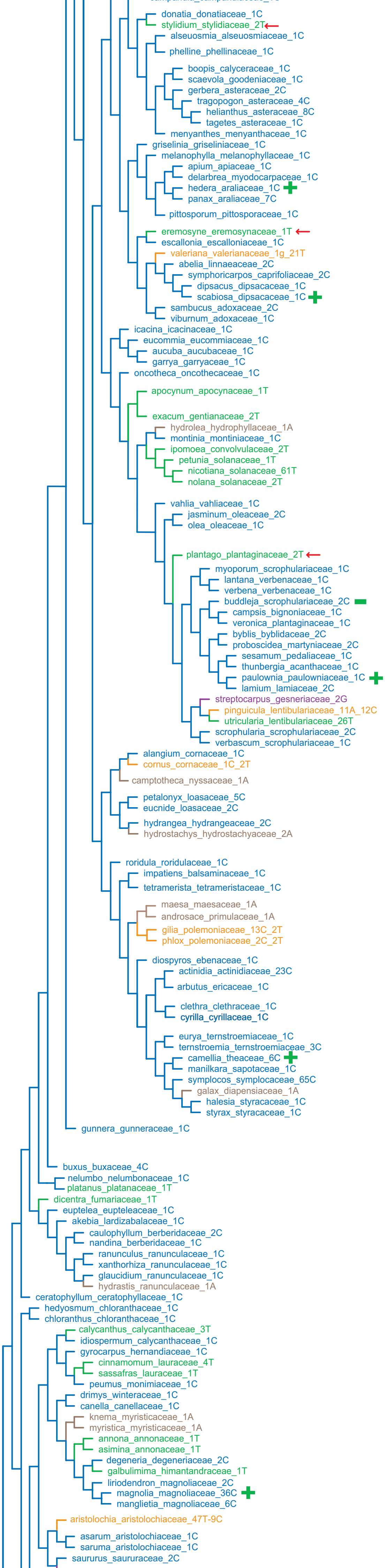
donatia_donatiaceae_1C
stylidium_stylidiaceae_2T
alseuosmia_alseuosmiaceae_1C
phelline_phellinaceae_1C
boopis_calyceraceae_1C
scaevola_goodeniaceae_1C
gerbera_asteraceae_2C
tragopogon_asteraceae_4C
helianthus_asteraceae_8C
tagetes_asteraceae_1C
menyanthes_menyanthaceae_1C
griselinia_griseliniaceae_1C
melanophylla_melanophyllaceae_1C
apium_apiaceae_1C
delarbrea_myodocarpaceae_1C
hedera_araliaceae_1C
panax_araliaceae_7C
pittosporum_pittosporaceae_1C
eremosyne_eremosynaceae_1T
escallonia_escalloniaceae_1C
valeriana_valerianaceae_1g_21T
abelia_linnaeaceae_2C
symphoricarpos_caprifoliaceae_2C
dipsacus_dipsacaceae_1C
scabiosa_dipsacaceae_1C
sambucus_adoxaceae_2C
viburnum_adoxaceae_1C
icacina_icacinaceae_1C
eucommia_eucommiaceae_1C
aucuba_aucubaceae_1C
garrya_garryaceae_1C
oncotheca_oncothecaceae_1C
apocynum_apocynaceae_1T
exacum_gentianaceae_2T
hydrolea_hydrophyllaceae_1A
montinia_montiniaceae_1C
ipomoea_convolvulaceae_2T
petunia_solanaceae_1T
nicotiana_solanaceae_61T
nolana_solanaceae_2T
vahlia_vahliaceae_1C
jasminum_oleaceae_2C
olea_oleaceae_1C
plantago_plantaginaceae_2T
myoporum_scrophulariaceae_1C
lantana_verbenaceae_1C
verbena_verbenaceae_1C
buddleja_scrophulariaceae_2C
campsis_bignoniaceae_1C
veronica_plantaginaceae_1C
byblis_byblidaceae_2C
proboscidea_martyniaceae_2C
sesamum_pedaliaceae_1C
thunbergia_acanthaceae_1C
paulownia_paulowniaceae_1C
lamium_lamiaceae_2C
streptocarpus_gesneriaceae_2G
pinguicula_lentibulariaceae_11A_12C
utricularia_lentibulariaceae_26T
scrophularia_scrophulariaceae_2C
verbascum_scrophulariaceae_1C
alangium_cornaceae_1C
cornus_cornaceae_1C_2T
camptotheca_nyssaceae_1A
petalonyx_loasaceae_5C
eucnide_loasaceae_2C
hydrangea_hydrangeaceae_2C
hydrostachys_hydrostachyaceae_2A
roridula_roridulaceae_1C
impatiens_balsaminaceae_1C
tetramerista_tetrameristaceae_1C
maesa_maesaceae_1A
androsace_primulaceae_1A
gilia_polemoniaceae_13C_2T
phlox_polemoniaceae_2C_2T
diospyros_ebenaceae_1C
actinidia_actinidiaceae_23C
arbutus_ericaceae_1C
clethra_clethraceae_1C
cyrilla_cyrillaceae_1C
eurya_ternstroemiaceae_1C
ternstroemia_ternstroemiaceae_3C
camellia_theaceae_6C
manilkara_sapotaceae_1C
symplocos_symplocaceae_65C
galax_diapensiaceae_1A
halesia_styracaceae_1C
styrax_styracaceae_1C
gunnera_gunneraceae_1C
buxus_buxaceae_4C
nelumbo_nelumbonaceae_1C
platanus_platanaceae_1T
dicentra_fumariaceae_1T
euptelea_eupteleaceae_1C
akebia_lardizabalaceae_1C
caulophyllum_berberidaceae_2C
nandina_berberidaceae_1C
ranunculus_ranunculaceae_1C
xanthorhiza_ranunculaceae_1C
glaucidium_ranunculaceae_1C
hydrastis_ranunculaceae_1A
ceratophyllum_ceratophyllaceae_1C
hedyosmum_chloranthaceae_1C
chloranthus_chloranthaceae_1C
calycanthus_calycanthaceae_3T
idiospermum_calycanthaceae_1C
gyrocarpus_hernandiaceae_1C
cinnamomum_lauraceae_4T
sassafras_lauraceae_1T
peumus_monimiaceae_1C
drimys_winteraceae_1C
canella_canellaceae_1C
knema_myristicaceae_1A
myristica_myristicaceae_1A
annona_annonaceae_1T
asimina_annonaceae_1T
degeneria_degeneriaceae_2C
galbulimima_himantandraceae_1T
liriodendron_magnoliaceae_2C
magnolia_magnoliaceae_36C
manglietia_magnoliaceae_6C
aristolochia_aristolochiaceae_47T-9C
asarum_aristolochiaceae_1C
saruma_aristolochiaceae_1C
saururus_saururaceae_2C

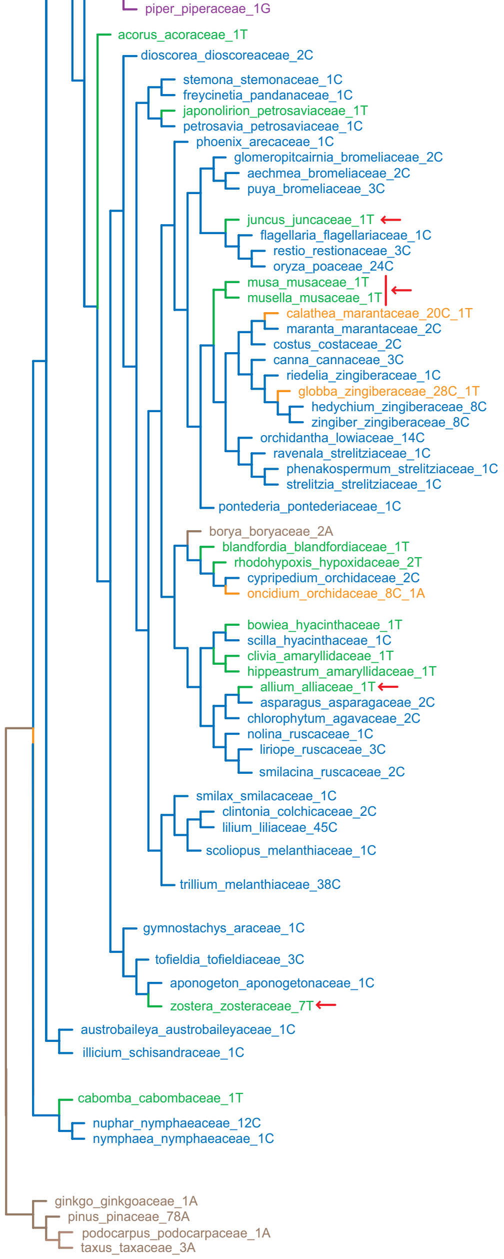

Evolution of *matK*-3 editing sites in angiosperms

Nucleotides found at the *matK*-3 editing site in angiosperms were mapped on a phylogenetic tree (Soltis et al. 2000). Four gymnosperms were used as an outgroup. Genus and family names are followed by a number indicating the number of species carrying a C or a T at the editing site (blue = purely C-containing lineages; T = purely T-containing lineages; brown = purely A-containing lineages; lilac = purely G-containing lineages; orange = mixed lineages). Several species were tested experimentally for processing of *matK*-3. (+/- = selected species of this genus does/does not edit site; red arrows = lineages that have independently lost the C at *matK*-3.)
