# Supplementary material for: Loss of matK RNA editing in seed plant chloroplasts
Source: BMC Evol Biol. 2009 Aug 13;9:201. doi: 10.1186/1471-2148-9-201 (PMC2744683; doi:10.1186/1471-2148-9-201)
Supplement: Additional file 3 — Examples of matK-2 editing sites lost during Angiosperm evolution. An excerpt of the phylogenetic tree shown in Additional file 1 labeled to demonstrate the method used to evaluate losses of editing sites during matK evolution in angiosperms. [file 1471-2148-9-201-S3.pdf]

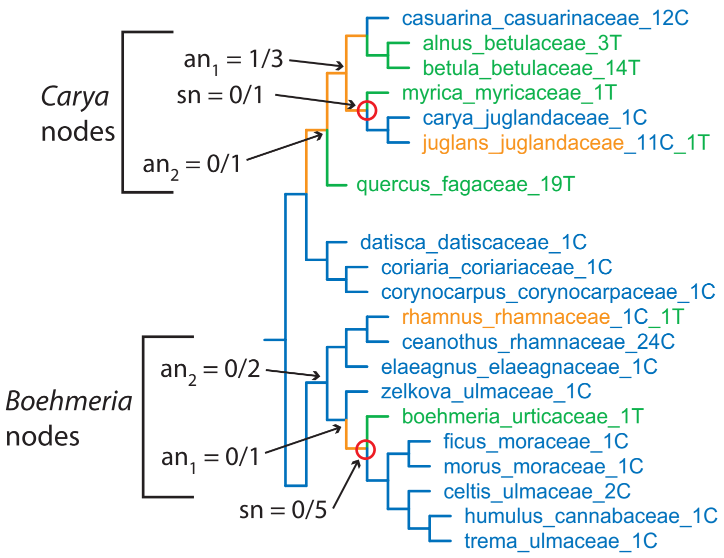

Examples of *matK*-2 editing sites lost during Angiosperm evolution.

RNA editing sites were identified in sequence alignments of *matK* and mapped onto a phylogenetic tree (Soltis et al. 2000). Shown is an excerpt of a larger tree (see Additional file 2). Genus and family names are followed by a number indicating the number of species carrying a C or a T at the editing site (blue = purely C-containing lineages; green = purely T-containing lineages; orange = mixed lineages). Two examples are given for the algorithm used to decide whether a nucleotide difference between adjacent lineages reflected an independent mutation. (sn = sister node; an = ancestral node; red circle = example nodes.) Numbers indicate how many genera at a node contain the same/different nucleotide from the genus analyzed (not including the analyzed genus itself or mixed genera). In *Boehmeria*, more than 80% of species in all three related nodes analyzed have a C, thus marking *Boehmeria* as a representative of an independent C-to-T mutation at *matK*-2. By contrast, *Carya* does not match these criteria for a T-to-C mutation at ancestral node 1.
