# Supplementary material for: Loss of matK RNA editing in seed plant chloroplasts
Source: BMC Evol Biol. 2009 Aug 13;9:201. doi: 10.1186/1471-2148-9-201 (PMC2744683; doi:10.1186/1471-2148-9-201)
Supplement: Additional file 4 — List of independent C-to-T losses in angiosperm evolution at matK editing sites. A table listing all C-to-T losses at matK editing sites identified in this study based on the analysis of the phylogenetic trees shown in Additional files 1 and 2. [file 1471-2148-9-201-S4.pdf]

List of independent C-to-T losses in angiosperm evolution at *matK* editing sites

Group	Genus[a]	Genera at sn (T/C) [b]	Genera at an1 (T/C) [b]	Genera at an2 (T/C) [b]	Mixed Genera		
					equal number of Ts and Cs	more Cs than Ts (C/T)	more Ts than Cs (T/C)
***matK*-2**							
Rosids	*Boehmeria*	0/5	0/1	0/2	*Rhamnus* (1/1)	*Juglans* (11/1)	
	Tropaeolum	1/7	2/17	0/1			
	Batis	0/1	0/3	0/2			
	Sterculia	0/3	0/1	0/1			
Asterids	*Helianthus/Tagetes*	0/1	0/1	0/2	*Plantago* (1/1)	*Utricularia* (25/1)	*Valeriana* (1/22)[c]
	Apium	0/1	0/2	0/1		*Gilia* (14/1)	
	Streptocarpus	0/1	0/2	0/12			
	Eurya/Ternstroemia	0/6	0/4	0/1			
Basal Eudicots	*Buxus*	17/87	0/1	1/9			
	Nandina	0/1	0/4	0/1			
Magnoliids	*Gyrocarpus*	0/3	0/2	0/11			
	Saururus	0/1	0/3	1/17			
**total**		**12**				**5**	**1**
***matK*-3**							
Rosids	*Coriaria*	0/1	0/7	1/13	*Reseda* (1/1)		
	Celtis	0/2	0/2	0/1			
	Ailanthus	0/2	0/4	0/1			
Saxifragales	*Crassula* branch (6 gen.)	0/2	0/2	0/2			
Caryophyllids	*Celosia* branch (5 gen.)	0/1	0/1	1/6			
Asterids	*Stylidium*	0/1	0/9	0/2	*Phlox* (2/2)	*Gilia* (13/2)	
	Eremosyne	0/1	0/7	0/7		*Cornus* (11/2)	
	Plantago	1/17	0/2	0/1			
Magnoliids						*Aristolochia* (47/9)	
Monocots	*Juncus*	1/3	1/3	2/13		*Calathea* (20/1)	
	Musa/Musella	0/10	0/1	1/7		*Globba* (28/1)	
	Allium	0/1	0/1	0/3			
	Zostera	0/1	0/1	0/1			
**total**		**12**				**7**	

[a] taxa containing only species with a T at *matK* editing site

[b] numbers summarize the genera at a node that contain only species with a T at the editing site versus those genera that contain a C at the editing site. sn = sister node; an1 and an2 = ancestral nodes 1 and 2; see suppl. Fig. 1 for further explanations

[c] The genus *Valeriana* is the only one that combines more species with a T at *matK*-2 than those with a C at a ratio of 22 to 1, which could potentially indicate a C-gain. The genus *Valeriana* is set in a background of C-containing taxa, the Campanulids. The *Valeriana* species carrying a C is *V. hardwickii,* a sister to the remainder of all other *Valeriana* species analysed here [40]. In fact, *V.*

hardwickii is considered to be outside of the *Valeriana* genus and thus belongs to the other C-carrying Campanulids [40]. Therefore, no T-to-C change has to be invoked for the taxon *Valeriana.*
