# Supplementary material for: Loss of matK RNA editing in seed plant chloroplasts
Source: BMC Evol Biol. 2009 Aug 13;9:201. doi: 10.1186/1471-2148-9-201 (PMC2744683; doi:10.1186/1471-2148-9-201)
Supplement: Additional file 5 — List of independent C-to-A and C-to-G losses in angiosperm evolution at the matK-3 editing site. A table listing all C-to-A and C-to-G mutations at matK editing sites identified in this study based on the analysis of the phylogenetic trees shown in Additional files 1 and 2. [file 1471-2148-9-201-S5.pdf]

List of independent C-to-A and C-to-G losses in angiosperm evolution at the *matK*-3 editing site

Group	Genus[a]	Genera at Sister Node (R/C)	Genera at Upper Node 1 (R/C)	Genera at Upper Node 2 (R/C)	Mixed Genera		
					equal number of Rs and Cs	more Cs than Rs	more Rs than Cs
**C to A**							
Rosids	*Populus*	0/1	0/2	0/1			
	Datisca	0/1	0/7	1/13			
	Spirea	0/3	0/9	1/9			
	Brassica	0/1	0/1	0/1			
	Vitis	0/1	4/47	1/11			
Saxifragales	*Itea*	0/1	0/1	0/2			
Caryophyllales	*Drosera branch*	0/1	0/2	0/1			
Asterids	*Hydrolea*	0/1	0/17	0/2		*Pinguicula* (12C/11A)	
	maesa/androsace	1/13	0/2	0/1			
	Galax	0/2	0/1	0/2			
Basal Eudicots	*Hydrastis*	0/1	0/2	0/2			
Monocots	*Knema/Myristica*	0/4	0/2	0/3			
	Borya	0/1	0/6	0/18			
**total**		**13**					
**C to G**							
	Piper	0/1	0/2	0/9		*Oncidium* (8/1)	
	Streptocarpus	0/2	0/12	0/2			
**total**		**2**				**2**	
