# Supplementary material for: Loss of matK RNA editing in seed plant chloroplasts
Source: BMC Evol Biol. 2009 Aug 13;9:201. doi: 10.1186/1471-2148-9-201 (PMC2744683; doi:10.1186/1471-2148-9-201)
Supplement: Additional file 6 — Analysis of matK-2 and matK-3 editing in selected species. Excerpts from cDNA sequencing electropherograms are shown to demonstrate the extent of editing in selected angiosperm species. [file 1471-2148-9-201-S6.pdf]

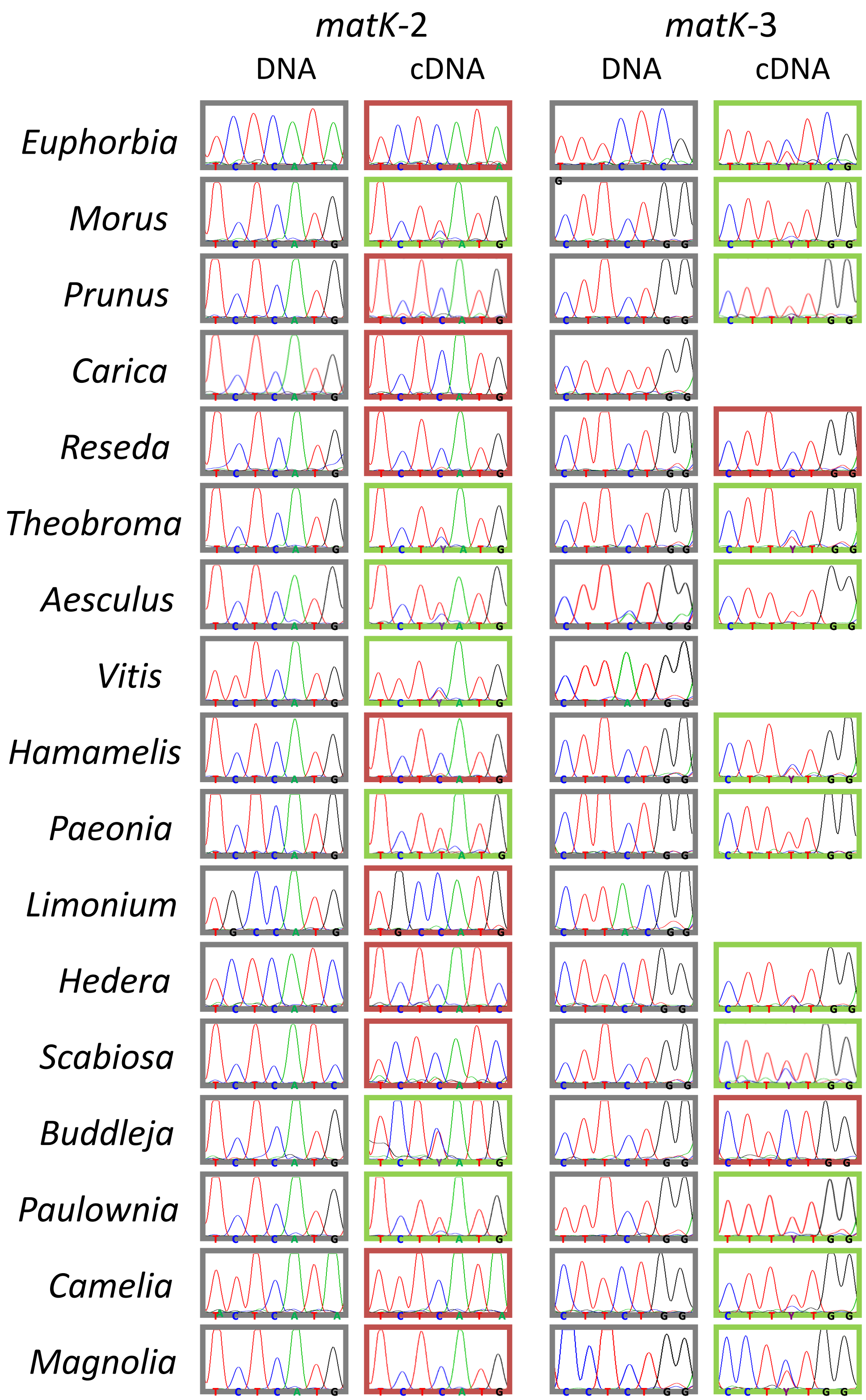

Analysis of *matK*-2 and *matK*-3 editing in selected species.

Sequence analysis of amplified *matK* cDNA and DNA for the species indicated on the left. Excerpts of sequence trace data are shown with the editing site at the center of seven sequenced bases. Frame color indicates editing status (green = site edited or partially edited; red = site remains unedited; gray = DNA sequence). These data were used for the analysis shown in Figure 2B.
